# Prevalence and molecular identification of *Cryptosporidium* isolates from pet lizards and snakes in Italy

**DOI:** 10.1051/parasite/2012194437

**Published:** 2012-11-15

**Authors:** L. Rinaldi, M. Capasso, A.D. Mihalca, R. Cirillo, G. Cringoli, S. Cacciò

**Affiliations:** 1 Department of Animal Pathology and Health, Faculty of Veterinary Medicine, University of Naples Federico II Via della Veterinaria 1 80137 Naples Italy; 2 Department of Parasitology and Parasitic Diseases, Faculty of Veterinary Medicine, University of Agricultural Sciences and Veterinary Medicine Cluj-Napoca Calea Manastur 3-5 Cluj-Napoca 400372 Romania; 3 Department of Infectious, Parasitic and Immunomediated Diseases, Istituto Superiore di Sanità viale Regina Elena 299 00161 Rome Italy

**Keywords:** *Cryptosporidium*, reptile, lizard, snake, *Python molurus*, Italy, *Cryptosporidium*, reptile, lézard, serpent, *Python molurus*, Italie

## Abstract

In order to acquire prevalence and genetic data on *Cryptosporidium* infections in captive lizards and snakes kept as pets, a survey was conducted on 150 individual reptiles from southern Italy. Fecal samples were preserved in 5% formalin and analyzed using a commercial immunofluorescence assay (IFA) for the detection of *Cryptosporidium* oocysts and *Giardia* cysts. IFA revealed the presence of *Cryptosporidium* oocysts in nine of the 150 samples examined (6.0%), precisely in 6/125 snakes (4.8%) and in 3/25 lizards (12.0%); all fecal samples tested negative for the presence of *Giardia* cysts. Molecular characterization based on nested PCR amplification and sequencing of the SSU-rRNA gene, revealed the presence of *Cryptosporidium serpentis* in three samples from snakes (*Boa constrictor constrictor*, *Elapheguttata guttata guttata* and *Python molurus*).

## Abstract (fr)

Afin de déterminer la prévalence et l’identification par génotypage des infections à *Cryptosporidium* chez les lézards et les serpents, animaux de compagnie, une enquête a été menée en Italie du sud sur 150 reptiles. Les échantillons fécaux ont été conservés dans du formol à 5 % et analysés à l’aide d’un test d’immunofluorescence (IF) du commerce, permettant la détection simultanée d’oocystes de *Cryptosporidium* et de kystes de *Giardia*. L’IF a montré la présence d’oocystes de *Cryptosporidium* dans neuf des 150 échantillons examinés (6,0 %), plus précisément chez 6/125 serpents (4,8 %) et 3/25 lézards (12,0 %). Tous les échantillons ont été négatifs pour la recherche des kystes de *Giardia*. La caractérisation moléculaire, basée sur l’amplification par PCR nichée et séquençage du gène SSU-rRNA a identifié *C. serpentis* dans trois échantillons fécaux de serpents (*Boa constrictor constrictor*, *Elaphe guttata guttata* et *Python molurus*).

In recent years, the number of reptiles kept as exotic pets in households has significantly increased (Copping, 2008). However, the acquisition of reptiles, either as captive bred or wild caught animals, yields a high risk of disease introduction into a collection and for this reason, reptilian medicine has evolved greatly in recent decades (Pasmans *et al.*, 2008). Stressful life, concentration of animals and the presence of different subjects in a small living space can promote the spreading of pathogens. Moreover, all these factors may suppress the immune response in reptiles and increase the opportunity for pathogens to cause infections and consequent diseases (Rataj *et al.*, 2011; Dipineto *et al.*, 2012) which can be spread to other individuals or other species, including humans. Therefore, an examination for endoparasites should be performed as part of the routine check of all reptiles and only parasitefree or successfully treated animals should be introduced into a healthy collection (Pasmans *et al.*, 2008).

*Cryptosporidium* infections are common in wild and captive reptiles (Upton *et al.*, 1989) and often establish as chronic and sometimes lethal infections (Pasmans *et al.*, 2008). Several *Cryptosporidium* species have been described from reptiles since the first report by Brownstein (1977). However, currently only two species are considered valid, namely *C. serpentis* and *C. varanii* (Fayer, 2008). *C. serpentis* is associated mainly with snakes and infects usually the gastric epithelium while *C. varanii* has intestinal predilection and is primarily associated with lizards (Xiao *et al.*, 2004). Recently, molecular evidence suggested the existence of a third species in tortoises (Traversa, 2010).

Snakes infected with *C. serpentis* may show symptoms of mild to severe gastritis with frequent regurgitation, particularly after feeding, while *C. varanii* causes enteritis and diarrhea. None of this reptile associated *Cryptosporidium* species is infective to mammals; hence their zoonotic importance is absent. However, oocysts of the zoonotic species *C. parvum* and *C. muris* have been also detected in the feces of captive snakes and lizards, and probably originate from infected mammals used as food by carnivorous reptiles (Xiao *et al.*, 2004; Pedraza-Díaz *et al.*, 2009; Richter *et al.*, 2011). The bovine genotype of *C. parvum* has been recently isolated also from wild tortoises in Romania (D’Amico *et al.*, unpublished data).

Despite the importance of *Cryptosporidium* infections in snakes and lizards, due to their impact on animal and public health and welfare, only few comprehensive surveys have been conducted on the prevalence and molecular characterization of *Cryptosporidium* isolates in captive reptiles (e.g. Pedraza-Díaz *et al.*, 2009; Richter *et al.*, 2011).

In this study, we performed a survey on the prevalence and molecular identification of *Cryptosporidium* in fecal samples from reptiles (snakes and lizards) kept as pets in the Campania region of southern Italy.

## Materials and Method

### Sampling and Immunofluorescence

From September 2009 to June 2010 individual fecal samples and cloacal swabs were collected from 150 captive reptiles (125 snakes and 25 lizards). Out of these 150 animals, 94 (62.7 %) were housed in exotic animal farms, nine (6 %) in pet shops and 47 (31.3 %) in private households. For each animal, anamnesis data (species, sex, age, micro-environment conditions, captive-born or wild-caught origin, feeding, cohabitation, gastro-intestinal symptoms, use of antiparasitic treatments) were recorded. All fecal samples were stored in 5 % formalin and subsequently examined in the laboratory using the FLOTAC Pellet Technique (Cringoli *et al.*, 2010) in order to detect parasitic and pseudoparasitic elements (for the results see Rinaldi *et al.*, 2012). In addition, on each fecal sample, a direct immunofluorescence assay (IFA) was used to investigate the presence of *Cryptosporidium* oocysts and *Giardia* cysts using a commercial kit (MERIFLUOR *Cryptosporidium*/*Giardia*, Meridian Bioscience Diagnostic, Cincinnati, OH, USA). Fecal suspension was concentrated with a Sucrose Solution (specific gravity = 1.200) and the sediment obtained was applied to slides for IF and stained according to the manufacturer’s instructions. Oocysts were identified and counted by fluorescence microscopy at 400 × on the basis of apple-green fluorescence and morphometric features (size, width and internal structures).

### Molecular Characterization

Prior to DNA extraction, fecal samples (200 µl) resulted positive for *Cryptosporidium* by IFA were washed three times with phosphate-buffered saline (pH 7.4) and centrifuged. DNA extraction was performed using a commercial kit (QIAamp DNA stool mini kit, Qiagen, Hilden, Germany).

A nested PCR protocol (Nichols *et al.*, 2003) was used for the amplification of a fragment of the small subunit ribosomal RNA gene (SSU-rRNA) of *Cryptosporidium* spp. In the primary PCR, a fragment of ~ 655 bp was amplified with the primers N-DIAGF2 (5’-CAA TTG GAG GGC AAG TCT GGT GCC AGC-3’) and NDIAGR2 (5’-CCT TCC TAT GTC TGG ACC TGG TGA GT-3’), whereas in the nested PCR reaction, a fragment of ~ 455 bp was amplified using the primers DIAGF (5’-AAG CTC GTA GTT GGA TTT CTG-3’) and DIAGR (5’-TAA GGT GCT GAA GGA GTA AGG-3’) originally described by Johnson *et al.* (1995). PCR conditions were as reported by Nichols *et al.* (2003). Amplification was performed in a Verity96 thermal cycler. Negative (water) and positive (DNA of *C. parvum*) controls were included in each experiment. Amplification products were separated by gel electrophoresis and visualized by ethidium bromide staining.

PCR products were purified using the Qiaquick purification kit (Qiagen, Milan, Italy) and sequenced on both strands using the ABI Prism BIGDYE Terminator Cycle Sequencing Kit (Life Technologies, Carlsbad, CA, USA) according to the manufacturer’s instructions. The sequencing reactions were analyzed using the ABI 3100 automatic sequencer (Applied Biosystems) and sequences were assembled using the software program SeqMan II (DNASTAR, Madison WI, USA).

## Results

All the 150 reptiles in this study were asymptomatic and didn’t receive any antiparasitic treatment. The diet of snakes included rodents, chickens and rabbits; lizards were fed mainly with vegetables and arthropods, depending on the species and size. A total of 125 snakes of 16 different genera (*Acanthophis*, *Aspidites*, *Boa*, *Bogertophis*, *Crotalus*, *Drymarcon*, *Elaphe*, *Gonyosoma*, *Hydrodynastes*, *Lampropeltis*, *Lamprophis*, *Lichanura*, *Morelia*, *Oreocryptophis*, *Python*, *Rhyncophis*) and 25 lizards of ten different genera (*Basiliscus*, *Chamaleo*, *Clamydiosaurus*, *Eublepharis*, *Iguana*, *Phelsuma*, *Physignatus*, *Pogona*, *Tupinambis*, *Zoonosaurus*) were examined.

The presence of *Cryptosporidium* oocysts was detected by IFA in nine out of the 150 samples examined (6.0 %; 95 % CI = 3.0-11.4 %), precisely in 6/125 snakes (4.8 %; 95 % CI = 2.0-10.6 %) and in 3/25 lizards (12.0 %; 95 % CI = 3.1-32.3 %) (Table I). In general, the number of oocysts observed was low, with most samples showing between one and ten oocysts per slide. All fecal samples tested negative for the presence of *Giardia* cysts. No significant association (P > 0.05 using the chi-squared test) was found between the origin of the reptiles (exotic farms, pets shop or private households) and the positivity to *Cryptosporidium*.

**Table I.** List of the reptile species that tested positive by IFA for the presence of *Cryptosporidium* oocysts.

			Positivity to *Cryptosporidium*		

Group	Common name	Scientific name	IF	PCR	*Cryptosporidium* species
Lizards	Veiled chameleon	*Chamaeleo calyptratus*	1	1	n.d.
	Frilled lizard	*Chlamydosaurus kingii*	1	1	n.d.
	Leopard gecko	*Eublepharis macularius*	1	n.a.	–

Snakes	Boa constrictor	*Boa constrictor constrictor*	1	1	*C. serpentis*
	Boa constrictor	*Boa constrictor imperator*	1	n.a.	–
	Corn snake	*Elaphe guttata guttata*	2	1	*C. serpentis*
	Indian rock python	*Python molurus*	2	1	*C. serpentis*

n.a. = not available for molecular characterization; n.d. = not identified at the species level.

Only five of the nine *Cryptosporidium*-positive samples were available for molecular characterization. Nested PCR amplification of the SSU-rRNA gene yielded the expected fragment (~ 450 bp) from three snake isolates (*Boa constrictor constrictor*, *Elaphe guttata guttata*, *Python molurus*) and sequencing revealed 100 % homology with *C. serpentis* (*e.g.*, GenBank accession number AF151376). In the case of lizards, weak amplification products were observed after the first PCR, but no amplification was obtained using nested PCR. The sequences of the first amplification products were of insufficient quality to identify the species.

## Discussion

*Cryptosporidium* infections are common in wild and captive reptiles; over 80 reptile species have been reported to be infected, including snakes, lizards and tortoises (Graczyk, 2008). The present study reports the results of the first IFA and molecular survey of *Cryptosporidium* in snakes and lizards in Italy. A recent coprological survey in pet reptiles in Italy (Papini *et al.*, 2011) showed *Cryptosporidium* infection in two leopard geckos (*Eublepharis macularius*) but molecular identification of the *Cryptosporidium* isolates to species level was not performed. In this study the presence of *Cryptosporidium* oocysts was detected by IFA in 9 samples (6.0 %), six from snakes and three from lizards. Molecular characterization by nested PCR amplification and sequencing of the SSU-rRNA gene demonstrates the presence of *C. serpentis* in the snakes *Boa constrictor*, *Elaphe guttata* guttata and *Python molurus*. It should be noted that *C. serpentis* have been previously reported in boa and corn snakes, however, according to the author’s knowledge, this is the first report of the presence of *Cryptosporidium* (*C. serpentis*) in *Python molurus* (Indian rock python). On the other hand, the *Cryptosporidium* parasites present in samples from lizards could not be identified by this approach. The weak amplification observed after the primary PCR reaction, and the lack of specific amplification in the secondary PCR reaction was mainly due to the preservation of the samples in formalin (see below) but could also suggest that DNAs from other organisms can bind PCR primers and reduce or prevent amplification of *Cryptosporidium* DNA.

Regarding *Giardia*, the present study confirmed the absence of this protozoan in snakes and lizards. Indeed, reptiles are not listed as potential hosts for parasites in the *Giardia* genus (Thompson *et al.*, 2011) and only one description of a *Giardia varani*-like flagellate from a water monitor, *Varanus salvator*, has been reported in Malaysia (Upton *et al.*, 1997).

This study added data to the few comprehensive surveys on the prevalence of *Cryptosporidium* in pet snakes and lizards in Europe (*e.g.* Pedraza-Díaz et al., 2009; Richter *et al.*, 2011). In agreement with previous studies (*i.e.* Pedraza-Díaz et al., 2009; Papini *et al.*, 2011), none of the infected animals showed signs of clinical cryptosporidiosis at the sampling time. The low numbers of oocysts detected as well as the absence of clinical signs may indicate a subclinical stage of infection, which can last for years in these animals (Pasmans *et al.*, 2008). Even if *Cryptosporidium* infection in reptiles is frequently chronic, *C. serpentis* infection in snakes may cause clinical disease (gastric hyperplasia, postprandial regurgitation and firm midbody swelling or chronic debilitating enteritis) and pathological changes. Currently, there are no effective control strategies against cryptosporidiosis in reptiles (Plutzer *et al.*, 2007).

*C. varanii* is the species most frequently detected in lizards causing weight loss and enteritis in leopard geckos, *Eublepharis macularius* (Taylor *et al.*, 1999; Terrell *et al.*, 2003; Deming *et al.*, 2008); in addition, cloacal prolapse and cystitis in green iguana (*Iguana iguana*) caused by a novel *Cryptosporidium* genotype has been also recently described (Kik *et al.*, 2011).

The aim of this survey was to obtain preliminary data on the prevalence of *Cryptosporidium* infection in snakes and lizards kept as pets in southern Italy; unfortunately, identification of *Cryptosporidium* species could not be obtained for all the IFA positive samples, probably because feces were fixed in formalin and this has an inhibitory effect on PCR amplification. Further studies deserve to be carried out in order to: (i) improve molecular identification; (ii) understand the source of *Cryptosporidium* infection for snakes and lizards; (iii) study in depth the real pathogenic effect of *C. serpentis*, *C. varanii* and other species; (iv) verify the possible occurrence of zoonotic *Cryptosporidium* species (as parasites or pseudo-parasites) in these new exotic pets.
